# From supportive care to strategic intervention: toward a multidisciplinary approach to cutaneous adverse events in cancer treatment

**DOI:** 10.1007/s00520-025-10128-1

**Published:** 2025-11-17

**Authors:** Davide Fattore, Michela Valeria Starace, Laura Serra-García, Antonin Schmitt, Florian Scotté

**Affiliations:** 1 Section of Dermatology, Department of Clinical Medicine and Surgery, University of Naples Federico II, Naples, Italy; 2 Dermatology Unit, IRCCS Azienda Ospedaliero-Universitaria Di Bologna, Bologna, Italy; 3 Department of Medical and Surgical Sciences, Alma Mater Studiorum University of Bologna, Bologna, Italy; 4 Dermatology Department, Hospital Clínic de Barcelona, Universitat de Barcelona, Barcelona, Spain; 5 Unicancer/UFR Des Sciences de Santé/INSERM U1231 - Center for Translational and Molecular Medicine - Oncology, Immunology and Epidemiology/Bureau, 379 - 7 Bd Jeanne dArc - BP, 87900 - 21079 Dijon Cedex, France; 6 Chef du Département Interdisciplinaire d’Organisation Des Parcours Patients (DIOPP), Université Paris Saclay, Paris, France

The landscape of cancer treatment has evolved dramatically in recent decades, shifting from traditional chemotherapy to more targeted and immunological approaches. These advancements have significantly improved treatment outcomes for many cancer types, offering hope for patients who previously had limited options. However, with these new therapies come distinct changes in the nature and profile of side effects, particularly dermatological manifestations [1]. Skin reactions to oncological treatments are common and have a negative impact on patient quality of life (QoL), leading to discomfort, disfigurement, and psychosocial distress [2]. Moreover, modern cancer therapies are often administered for prolonged periods, sometimes for the duration of the patient’s life, meaning that even mild dermatological issues can have lasting consequences. In addition, patients may interrupt or completely stop anticancer treatment because of skin toxicities. Consequently, these skin-related side effects can no longer be overlooked or relegated to secondary concerns.

The European Society for Medical Oncology (ESMO) has published symptom-oriented Clinical Practice Guidelines for the prevention and management of specific dermatological toxicities related to anticancer agents [2]. However, based on a review of the literature, there are still very few publications addressing the prevention of cutaneous toxicities. General recommendations applicable to all patients are lacking, the level of evidence remains low (Grades IV–V), and most proposals are symptom-oriented.

Regarding the treatment of cutaneous toxicities, there is a growing body of published literature from various organizations [2–5]. Consensus-based disease definitions have also been published to support the diagnosis of dermatological adverse events in patients treated with immunotherapy, but the level of evidence is low and more prospective clinical trials are needed [6]. In addition, the ongoing introduction of novel anticancer drugs, each with distinct toxicity profiles, further complicates the creation of a standardized protocol for oncology patients. These limitations highlight the urgent need for a multidisciplinary approach in developing evidence-based guidelines for the prevention and treatment of cutaneous toxicities, with the goal of improving patient well-being and QoL.

Oncologists, patients, and caregivers need to be educated on how to detect alarm signs for symptoms that require referral to a dermatologist.

Artificial intelligence (AI) is also likely to play a key role in the future management of side effects associated with anticancer treatment by predicting which patients are more prone to developing specific adverse effects, tailoring specific and individualized treatments, and possibly guiding the selection of anticancer treatments. AI may also play a role in monitoring skin toxicities in cancer patients and supporting them in the proper management of side effects [7, 8].

Importantly, patients and caregivers must be involved in all aspects of the prevention and management of cutaneous toxicities associated with oncological treatments. Consequently, it is also critical to translate clinical guidelines into useful material that is presented in validated, patient-friendly language. This represents a new approach, and healthcare professionals are likely to modify their practice because patients will ask them for support. If patients are aware of solutions for preventing and managing skin toxicity associated with oncology treatment, they will feel more empowered to ask their healthcare professionals the right questions.

From the patient’s perspective, avoiding skin toxicities firsthand is crucial. Providing information and advice before anticancer treatment can help reduce side effects. Community pharmacists are often the first healthcare professionals that patients interact with, but they lack sufficient information to offer relevant advice. Therefore, there is an opportunity to educate them about possible skin reactions to specific oncology drugs so they can share this information with patients.

In a recent patient survey that was conducted in France [9], 99% of 239 respondents said that they experienced skin toxicity associated with anticancer treatment and 59% of patients said that the toxicities impacted their everyday life. In addition, 93% of patients said that skin products (e.g., dermocosmetics, skin hygiene/makeup products) that they were using prior to anticancer treatment were not good enough to continue with after treatment, indicating that the skin is affected by the anticancer treatment and that the patients had to change their daily skincare routine because of oncology drug therapy. Patients need validated advice about the most appropriate skin products to use. Based on the survey results, this is clearly a common requirement among patients. Notably, the survey also showed that 80% of patients experienced an increase in their budget for dermocosmetics during treatment with oncology drugs [9].

The survey findings indicate that, even before taking into consideration the prevention and management of cutaneous toxicities, cancer patients receiving treatment experience a change in their normal skin condition that requires a different management approach. It is important to note that common dermatological side effects, such as pruritus and dry skin, can be prevented effectively with the use of therapeutic dermocosmetics (e.g., emollients). These products can support skin health during cancer treatment and can help manage and alleviate discomfort, improving the patient’s overall QoL.

When patients experience cutaneous toxicities of oncological treatment, it is essential that they receive timely advice from healthcare professionals on the best management strategy. Importantly, most of the advice and documentation that is currently available for managing cutaneous toxicities is prepared for women but, in reality, 50% of cancer patients are men. A more balanced approach is needed considering gender differences, including documentation that is relevant for transgender individuals.

There remains a lack of multidisciplinary approaches to oncology patients and a shortage of experts in oncodermatology. This gap in specialized care can hinder the timely and effective management of dermatological side effects, which are often overlooked or inadequately addressed [10]. A multidisciplinary approach is necessary to provide the best supportive care for cancer patients; better QoL is important to allow optimal efficacy for anticancer treatments. It is important to disseminate the right messages and information and to implement guidelines to improve daily clinical practice.

We have formed a European-focused Advisory Board representing the perspectives of oncologists, pharmacists, dermatologists, and patients, regarding the therapeutic dermocosmetic management of cutaneous adverse events associated with oncology treatments. Several potential future projects are currently under consideration, with the primary objective being the development of multidisciplinary consensus guidelines for the prevention of dermatologic toxicities in cancer patients, including both general recommendations and therapy-specific guidance.

## Data Availability

No datasets were generated or analysed during the current study.

## References

[1] Huynh Dagher S, Blom A, Chabanol H, Funck-Brentano E (2021) Cutaneous toxicities from targeted therapies used in oncology: literature review of clinical presentation and management. Int J Womens Dermatol 7(5Part A):615–624. 10.1016/j.ijwd.2021.09.009 35024416 10.1016/j.ijwd.2021.09.009 PMC8721134

[2] Lacouture ME, Sibaud V, Gerber PA, van den Hurk C, Fernández-Peñas P, Santini D et al (2021) Prevention and management of dermatological toxicities related to anticancer agents: ESMO clinical practice guidelines. Ann Oncol 32(2):157–170. 10.1016/j.annonc.2020.11.005 33248228 10.1016/j.annonc.2020.11.005

[3] Haanen J, Obeid M, Spain L, Carbonnel F, Wang Y, Robert C et al (2022) Management of toxicities from immunotherapy: ESMO clinical practice guideline for diagnosis, treatment and follow-up. Ann Oncol 33(12):1217–1238. 10.1016/j.annonc.2022.10.001 36270461 10.1016/j.annonc.2022.10.001

[4] Apalla Z, Nikolaou V, Fattore D, Fabbrocini G, Freites-Martinez A, Sollena P et al (2022) European recommendations for management of immune checkpoint inhibitors-derived dermatologic adverse events. The EADV task force ‘Dermatology for cancer patients’ position statement. J Eur Acad Dermatol Venereol 36(3):332–350. 10.1111/jdv.17855 34910332 10.1111/jdv.17855

[5] National Comprehensive Cancer Network (NCCN) Clinical Practice Guidelines in Oncology (NCCN Guidelines®). Management of immunotherapy-related toxicities. Version 1.2024 – 7 December 2023. Available from NCCN.org 10.6004/jnccn.2022.0020 35390769

[6] Chen ST, Semenov YR, Alloo A, Bach DQ, Betof Warner A, Bougrine A et al (2024) Defining D-irAEs: consensus-based disease definitions for the diagnosis of dermatologic adverse events from immune checkpoint inhibitor therapy. J Immunother Cancer 12(4):e007675. 10.1136/jitc-2023-007675 38599660 10.1136/jitc-2023-007675 PMC11015215

[7] Fan K, Cheng L, Li L (2021) Artificial intelligence and machine learning methods in predicting anti-cancer drug combination effects. Brief Bioinform 22(6):bbab271. 10.1093/bib/bbab271 34347041 10.1093/bib/bbab271 PMC8574962

[8] Burnette H, Pabani A, von Itzstein MS, Switzer B, Fan R, Ye F et al (2024) Use of artificial intelligence chatbots in clinical management of immune-related adverse events. J Immunother Cancer 12(5):e008599. 10.1136/jitc-2023-008599 38816231 10.1136/jitc-2023-008599 PMC11141185

[9] Chabanol H, Guéroult-Accolas L (2023) Impact des effets secondaires cutanes sur le quotidien des patients: leur pont du vue. 27 September 2023. Available from: https://www.patientsenreseau.fr/wp-content/uploads/2023/09/POSTERTOXCUTANEESAFSOS.pdf. Accessed 31 Mar 2025

[10] Fattore D, Lauletta G, Apalla Z, Sibaud V, Freites-Martinez A (2025) Dermatologic immune-related adverse events: it is time for a game change! J Eur Acad Dermatol Venereol 39(3):e271–e272. 10.1111/jdv.20282 39096035 10.1111/jdv.20282

